# Binder-Less Molybdenum Doped CoO Based Integrated Electrodes Fabricated by Electric Discharge Corrosion for High-Efficiency Supercapacitors

**DOI:** 10.3390/ma18010080

**Published:** 2024-12-27

**Authors:** Ri Chen, Zehan Xu, Yunying Xu, Tujun Lei, Dawei Liu, Chunlong Chen, Wenxia Wang, Igor Zhitomirsky, Muchao Qu, Guoying Zhang

**Affiliations:** 1 Department of Mechatronic Engineering, Guangdong Polytechnic Normal University, Guangzhou 510665, China; rchen01@gpnu.edu.cn (R.C.); xuzehan@gpnu.edu.cn (Z.X.); leitujunltj@163.com (T.L.); chenchunlongya@163.com (C.C.); 2 School of Education, Guangdong Polytechnic Normal University, Guangzhou 510665, China; yunying.xu@gpnu.edu.cn; 3 Department of Biomedical and Pharmaceutical Sciences, Guangdong University of Technology, Guangzhou 510006, China; fewwxia@gdut.edu.cn; 4 School of Materials Science and Engineering, McMaster University, Hamilton, ON L8S 4L7, Canada; zhitom@mcmaster.ca; 5 School of Automobile and Transportation Engineering, Guangdong Polytechnic Normal University, Guangzhou 510450, China; muchaoqu@hotmail.com

**Keywords:** electric discharge corrosion, molybdenum doped CoO, pulse width, binder-less

## Abstract

Due to its low cost, natural abundance, non-toxicity, and high theoretical capacitance, cobalt oxide (CoO) stands as a promising candidate electrode material for supercapacitors. In this study, binder-less molybdenum doped CoO (Mo@CoO) integrated electrodes were one-step fabricated using a simple electric discharge corrosion (EDC) method. This EDC method enables the direct synthesis of Mo@CoO active materials with oxygen vacancy on cobalt substrates, without any pre-made templates, conductive additives, or chemicals. Most importantly, the EDC method enables precise control over the discharge processing parameter of pulse width, which facilitates tailoring the surface morphologies of the as-prepared Mo@CoO active materials. It was found that the fabricated Mo@CoO based symmetric supercapacitor prepared by a pulse width of 24 μs (Mo@CoO-SCs24) achieved a maximum areal capacitance 36.0 mF cm^−2^ (0.15 mA cm^−2^), which is 1.83 and 1.97 times higher than that of Mo@CoO-SCs12 and Mo@CoO-SCs36. Moreover, the Mo@CoO-SCs24 devices could be worked at 10 V s^−1^, which demonstrates their fast charge/discharge characteristic. These results demonstrated the significant potential of the EDC strategy for efficiency fabricating various metal oxide binder-less integrated electrodes for various applications, like supercapacitors, batteries and sensors.

## 1. Introduction

With the rapid growth of the economy over the world and the substantial demand for fossil energy, an energy crisis and related environmental issues have emerged. It is extremely necessary to develop sustainable, clean, and cost-effective high-efficiency energy storage technologies [1,2,3,4,5]. Eco-friendly energy storage systems have been rapidly increased in number and considered as sustainable solutions for electronic devices, as in smart electronics, energy harvesting, hybrid–electric automobiles, and large energy storage system [6,7,8,9,10]. In diverse energy storage systems, supercapacitors have attracted considerable attention because of their high specific power, extended service life, environmental friendliness, and swift charge–discharge capabilities [11,12,13,14,15]. Supercapacitors can be primarily divided into two main types according to the mechanism for storing energy: electric double layer capacitors (EDLCs) and pseudo-capacitors [16,17,18]. EDLCs store energy by accumulating charge at the electrode–electrolyte interface without involving chemical reactions, which provides excellent cycle stability. However, the capacitance of EDLCs remains limited [19,20,21]. On the contrary, the energy storage of pseudo-capacitors, based on their redox reaction, happens on or near the electrode surface. This process allows for higher capacitance than that pf EDLCs by enabling greater active material involvement in charge storage [22,23,24]. Among various pseudocapacitive materials, transition metal oxides, favored for their surface faradic reaction and high capacitive performance, offer significant potential for supercapacitors [25]. Consequently, various metal oxides, such as RuO_2_ [26,27,28], TiO_2_ [29,30], ZnO [31,32,33], WO_3_ [34,35,36], Nb_2_O_5_ [37,38,39,40], MnO_2_ [41,42,43], Cu_2_O [44,45], CuO [46,47,48], NiO [49,50,51,52], MoO_x_ [53,54], FeO_x_ [55,56,57], VO_0.2_ [58], V_2_O_5_ [59,60,61], Co_3_O_4_ [62,63,64] and CoO [65,66,67,68], have been extensively investigated for the fabrication of supercapacitor electrodes. CoO exhibits relatively high conductivity compared to many other metal oxides. Moreover, it shows high capacitance, enhanced stability, cost-effectiveness, and excellent compatibility with other components, which makes it a promising candidate for supercapacitor electrodes [65,69,70,71]. However, its conductivity is still insufficient to meet the requirements for high-performance supercapacitors [69,70,72,73]. Therefore, in order to enhance the capacitive behavior of electrodes, a large amount of effort has focused on tthe introduction of metal element doping, micro-nano structures design, vacancies creation and composite design [70,74,75]. The introduction of foreign metal element doping had been proven to be an efficient strategy for modulating the electronic structure of transition metal oxides and thus enhancing electronic conductivity [76,77,78]. Pandey et al. studied the impact of magnetic fields and Al^3+^ doping on NiCoCuCH electrodes, which achieved enhanced redox activity and capacitance. Their optimized electrode at 110 mT delivered 1100 C g^−1^ at 2 A g^−1^, with 250 Wh kg^−1^ energy density and 1.7 kW kg^−1^ power density in an asymmetric device [79]. Thalji et al. employed a solvothermal method to grow Co-doped W_18_O_49_ on carbon cloth, which achieved a specific capacitance of 792 F g^−1^ at 1.0 A g^−1^, nearly twice that of the undoped material, due to enhanced meso-porosity and conductivity [80]. Xia et al. grew Mo-doped CoO nanosheets (Mo@CoO) on nickel foam as a novel non-enzyme electrode material, which proved that the introduction of Mo doping could effectively regulate surface defects and provide more active sites for energy storage [81]. Feng et al. fabricated CoO electrode materials decorated by oxygen vacancies, as well as copper doping (O_v_-Cu-CoO), using a hydrothermal method, followed by the processing of calcination. The combined effects of copper doping and oxygen vacancies synergistically adjust the conductive network of CoO, which in turn enhances its conductivity and significantly improves its electrochemical performance. The testing results showed that the O_v_-Cu-CoO electrode achieved a capacitance value of 1388.6 F g^−1^ at 1 A g^−1^ and retained an impressive capacitance retention of 81.2% at 20 A g^−1^ [65]. Zhou et al. synthesized Ni-doped CoO nanoparticles via a hydrothermal method. This fabricated Ni-doped CoO based electrode achieved an enhanced specific capacitance (270.6 F g^−1^ at 2 mV s^−1^) and a remarkable retention (106% after 500 cycles) [82]. These great improvements in electrochemical performance were due to the enhanced electronic conductivity and optimized electronic structure induced by the doping elements and oxygen vacancy, which facilitate faster electron transport and increase the availability of active sites for charge storage.

The fabrication of binder-less electrodes is an effective approach to enhance electrochemical performance [83,84,85]. Conventional electrode fabrication methods often require binders, which unavoidably increase costs and resource consumption. Organic binders have low electrical conductivity and low density. Therefore, even a small mass percentage of binder can result in a significant volume fraction for the insulating binder and low conductivity of the electrode. Therefore, avoiding the use of the insulating binder is beneficial for the fabrication of electrodes and devices with low impedance and enhanced performance. Furthermore, binder-less methods, like EDC, enable the direct deposition of active materials onto substrates, ensuring robust adhesion and excellent electrical contact between the electrode material and the current collector. This approach is scalable and well-suited for industrial applications, providing a cost-effective and environmentally friendly alternative to traditional binder-based methods. The morphology of the active material is relatively porous, which is beneficial for good electrolyte access to the active material. The loading density of the active material can be modified by the variation of EDC processing parameters, such as processing voltage, processing current, and pulse width. These processing parameters hold potential for achieving higher active mass loading and improved performance [74,84,86,87]. The reduction in the overall size of the electrode, facilitated by the binder-free approach, also improves the volumetric performance of energy storage devices. Various effective methods for fabricating binder-less electrodes have been reported, including electrostatic spray deposition (ESD), chemical vapor deposition (CVD), electrodeposition, magnetron sputtering an. ESD is a rapid and straightforward method for achieving binder-free deposition with precise morphology control. Similarly, CVD enables the growth of thin films directly on substrates, offering excellent adhesion and uniformity. Magnetron sputtering is employed for depositing high-quality thin films, offering precise control over film thickness and composition. Electrodeposition, on the other hand, is highly scalable, allowing for the direct deposition of active materials onto current collectors. For example, Adelowo et al. employed ESD to fabricate binder-free rGO-CNT electrodes, achieving lithium-ion capacitors with energy densities of 114.5 Wh kg^−1^ and power densities of 2569 W kg^−1^ [88]. Lu et al. used CVD to grow CNF on copper foam, followed by cobalt coating via electrochemical deposition, which was then oxidized by heating to form a Co@CoO/CNF/copper foam integrated electrode. This electrode achieved 19.2 F cm^−3^ at 50 mA cm^−3^ and showed a remarkable cyclic life of 97.5% (5000 cycles) [67]. Chen et al. utilized electrodeposition to grow graphene nanosheets onto Co/CoO core-shell nanostructures, creating a 3D binder-less Co/CoO/rGO electrode. The 3D interconnected binder-less electrode obtained 7.765 F cm^−2^ at 1 mA cm^−2^, which is because of the enhancement in the electron transport [89]. Wang et al. deposited a CoO buffer layer onto copper foil via magnetron sputtering following by uniformly grew CoO nanosheets on the film using a hydrothermal method. The CoO based integrated electrodes achieved a first-cycle discharge capacity of 1261 mAh g^−1^ at 200 mA g^−1^, maintained at 814.7 mAh g^−1^ after cyclic testing of 100 cycles [90]. These methods typically entail intricate preparation procedures and are time consuming, which pose challenges for scalability in large-scale fabricating and applications. Furthermore, these approaches might entail the utilization or generation of hazardous substances and require the aid of additional chemicals, resulting in waste generation and environmental concerns. Therefore, it is especially important to propose a simple, green and digital method for the fabrication of binder-less integrated electrodes for supercapacitors.

EDC is a rapid machining technique based on the electric–thermal effect, widely utilized for fabricating various alloys and advanced composite materials [91,92,93]. It was reported that the surface morphology of metallic plates can be tailored by adjusting the pulse width during EDC processing [94,95,96]. The ability to precisely control this EDC pulse width has significant implications for the customization of material properties. However, there is no related report on investigating the effect of EDC pulse width on the electrochemical performance of supercapacitors. Therefore, investigating the relationship between EDC pulse width and capacitive behavior of supercapacitor devices is essential to push forward the development of high-performance supercapacitors and related energy storage devices. For the first time, the effects of EDC pulse width on supercapacitor performance have been systematically investigated. This approach paves a new avenue for the one-step fabrication of high-performance metal oxide supercapacitor with tailored electrochemical properties in an automatic, efficient and green manner.

In this study, a simple electric discharge corrosion (EDC) method was employed to fabricate binder-less Mo doped CoO (Mo@CoO) electrodes for a symmetric supercapacitor. This EDC technique is a rapid method for precisely fabricating binder-less integrated electrodes directly on the metal substrates, without the need for pre-made templates or conductive additives, and without the aid of additional chemicals. During the discharge processing, a larger amount of heat was generated between Mo electrode wire and cobalt metal substrate, which is able to make the Mo wire and cobalt metal sheet melt. When the discharge processing is off, the molten materials can be quickly resolidified by the cooling effect of the deionized water, and subsequently the Mo doped CoO decorated with oxygen vacancy is formed directly on the Co metal substrate, which plays the part of current collector. The Mo@CoO integrated electrodes were one-step prepared without requiring any binders. Moreover, the simultaneous introduction of Mo element doping and oxygen vacancies in Mo@CoO is beneficial for the enhancement of its electronic conductivity, and thus greatly boost the overall electrochemical performance. These Mo@CoO integrated electrodes were then assembled into a symmetric supercapacitor device named Mo@CoO-SC. Notably, the EDC method allows for customizable discharge processing parameters, and a detailed study was conducted on the electrochemical performance based on varying pulse widths. The results showed that Mo@CoO-SCs24 achieved the largest areal capacitance of 36.0 mF cm^−2^ at 0.15 mA cm^−2^. These results demonstrated the significant potential of EDC strategy for the efficient fabrication of various metal oxide-based binder-less integrated electrodes for various applications, like supercapacitors, sensors and batteries.

## 2. Materials Sources and Methods

### 2.1. Materials Sources and Samples Preparation

High-purity Cobalt (Co) foils, with a purity of 99.99%, were procured from Qing-he-li-sheng Metal Materials Co. Ltd. (Xingtai, China) Potassium hydroxide was obtained from the Kell-chemical-technology corporation (Guangzhou, China). Polybdenum machining wires were purchased from Jin-dui-cheng Molybdenum Mine Bright (Shandong) Co. Ltd. (Zibo, China). Co foils underwent electric discharge corrosion using a computer-controlled electrical discharge machining apparatus (HB 400C). The Mo wire served as the machining tool, while the cobalt metal substrate played the role of workpiece. The wire cutting machine worked with the following parameters: a discharge voltage of 60 V, discharge current of 2 A, and a duty cycle of 4. The Mo@CoO based symmetric supercapacitors assembled with the Mo@CoO integrated electrode prepared with different EDC pulse widths of 12, 24, 36 and 48 μs were designated as Mo@CoO-SCs12, Mo@CoO-SCs24, Mo@CoO-SCs36, and Mo@CoO-SCs48, respectively.

### 2.2. Characterization Methods for Electrode Materials

X-ray photoelectron spectroscopy (XPS) analysis of the Mo@CoO active material was performed by the Thermo-scientific K-Alpha spectrometer (Thermo-scientific, Waltham, MA, USA). The surface morphology of the Mo@CoO active materials was studied by scanning electron microscope (SEM) (TESCAN, Brno, Czech Republic) equipped with the TESCAN, MIRA LMS model. Furthermore, the elemental distribution of the Mo@CoO electrode was investigated by employing energy-dispersive X-ray spectroscopy (EDS). To further investigate the vibrational modes and structural characteristics, Raman spectroscopy was conducted using the WITec alpha300R system (WITec GmbH, Ulm, Germany). Oxygen vacancies within the material were analyzed via an electron paramagnetic resonance (EPR) using the Bruker EMXplus-6/1 spectrometer (Bruker, Billerica, MA, USA). Finally, X-ray diffraction (XRD) analysis with a Rigaku SmartLab SE (Rigaku, Akishima-shi, Tokyo) was employed to compare the crystal structure and phase composition of the Co substrate and the Mo@CoO electrode.

### 2.3. Electrochemical Characterization

The electrochemical properties of Mo@CoO symmetric supercapacitors were investigated by the utilization of the CHI 660E workstation in a two-electrode system. The capacitive performance of Mo@CoO symmetric devices was investigated by cyclic voltammetry (CV), galvanostatic charging/discharging (GCD) and electrochemical impedance spectroscopy (EIS) techniques. The charge storage mechanism in KOH solutions can involve adsorption of OH^−^ groups on the particle surface and following surface reactions [97,98]:

In the negative potential range: CoOOH + e^−^ ⟷ CoO +OH^−^

In the positive potential range: CoOOH + OH^−^ ⟷ CoO_2_ + H_2_O +e^−^

## 3. Results and Discussion

**Figure 1:**
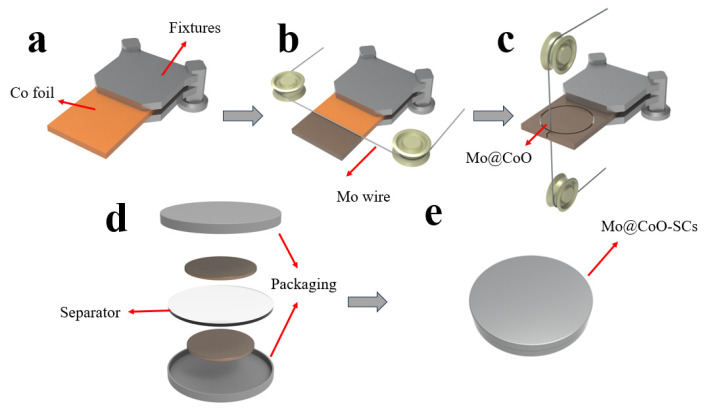
The process of fabricating binder-less Mo@CoO integrated electrodes and Mo@CoO-SCs using EDC technology. (**a**–**c**) Fabrication procedures for binder-free Mo@CoO integrated electrodes, and (**d**–**e**) assembly process for Mo@CoO-SCs.

**Figure 2:**
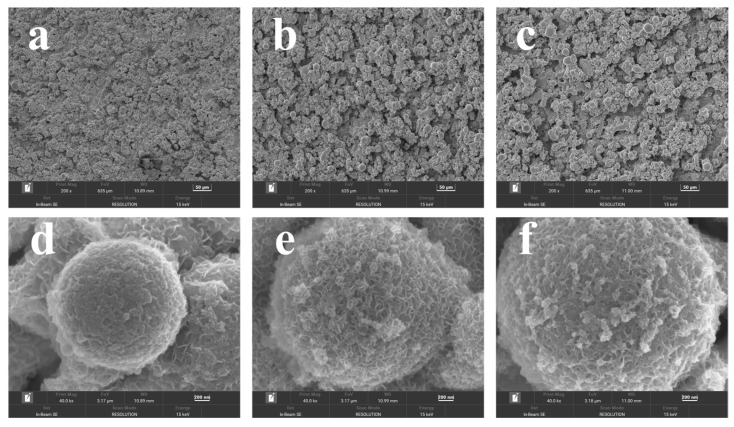
SEM pictures for Mo@CoO integrated electrodes prepared using EDC with different pulse widths: (**a**) Mo@CoO-12, (**b**) Mo@CoO-24, and (**c**) Mo@CoO-36, and their high-resolution counterparts (**d**) Mo@CoO-12, (**e**) Mo@CoO-24, and (**f**) Mo@CoO-36.

**Figure 3:**
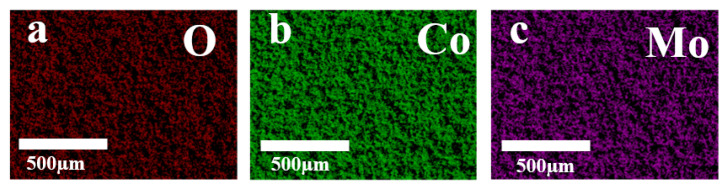
EDS elemental mapping of the Mo@CoO surface for (**a**) O, (**b**) Co, and (**c**) Mo.

Figure 1 showed the fabricating procedures for binder-less Mo@CoO integrated electrodes using EDC technology and assembling procedures for Mo@CoO symmetric supercapacitors. A smooth cobalt foil was chosen as the workpiece, where the EDC technique employed a molybdenum electrode wire as the machining tool to generate electric sparks between the Mo wire and the cobalt foil. During the discharge process, a larger amount of heat was generated between Mo electrode wire and cobalt metal substrate, which can make the Mo wire and cobalt metal sheet melt. When the discharge processing is off, the molten materials can be quickly resolidified by the cooling effect of the deionized water, and subsequently the Mo doped CoO decorated with oxygen vacancy was formed directly on the Co metal substrate, which played the part of current collector. Subsequently, the Mo@CoO-SCs based symmetric devices were assembled. The SEM result (Figure S1) demonstrated that the surface of the Co metal substrate before EDC was smooth. The raw Co metal substrate is not a pseudocapacitive material. The ooxidation of Co metal substrate is critically important for the formation of pseudocapacitive materials with a mixed Co and Mo oxide layer. The EDC process results in changing of the surface morphology, which is shown in Figure 2. Figure 2a–c displays the SEM images of Mo@CoO-12, Mo@CoO-24, and Mo@CoO-36 electrodes fabricated using EDC with varying pulse widths of 12, 24, and 36 μs, respectively. Differences in microstructural features could be clearly observed, which reflects that the applied pulse width using the EDC technique had an important effect on the surface morphology of the Mo@CoO integrated electrode. The increase in particle size with the rise in pulse duration from 12 to 36 μs could be attributed to the prolonged exposure to high-energy electric discharges during the EDC process. Longer pulse duration results in extended heating cycles, allowing more material to melt, coalesce, and subsequently re-solidify into larger particles. The Mo@CoO-12 sample prepared by a pulse width of 12 μs exhibited smaller particles with a sparse distribution on the substrate, while the Mo@CoO-36 sample showed significantly larger particles with noticeable aggregation, which could provide a limited surface area for energy storage. In contrast, the Mo@CoO-24 sample features moderately sized particles with uniform and porous cobalt oxide particles across the Co foil. Additionally, high-resolution SEM images have been provided in Figure 2d–f, offering additional information on the surface morphology of Mo@CoO electrodes prepared with different pulse widths. Compared to the other two samples of Mo@CoO-12 and Mo@CoO-36, the unique nanostructures of Mo@CoO-24 particles indicate a relatively higher specific surface area, providing abundant surface-active sites that enhance ion adsorption, promote effective electrolyte interaction, and support faster surface Faradaic redox reactions. Moreover, EDS mapping was carried out to examine the element distributed characteristic of the Mo@CoO electrode. The examination results indicated the even distribution characteristic of molybdenum, cobalt and oxygen on the surface of the cobalt foil, which confirmed that the cobalt foil has been effectively oxidized and the Mo element has been successfully introduced into the oxide layer generated on the cobalt foil surface during the machining process of EDC (Figure 3).

XRD was utilized to further elucidate the phase, crystalline structure and crystallinity of the Mo@CoO electrode. As shown in Figure S2, peaks at 2θ values of 41.8°, 44.7°, 47.7°, 62.8°, and 75.8°, observed in the Co substrate, are attributed to the (100), (002), (101), (102), and (110) planes of Co (JCPDS#05-0727) [99]. In contrast, the XRD patterns of Mo@CoO samples reveal the formation of a new Co phase, with peaks at 44.1°, 51.5°, and 75.6° corresponding to the (111), (200), and (220) planes of Co (JCPDS#15-0806), respectively [100,101]. No obvious difference was observed in XRD profiles for the samples prepared by EDC method with different pulse widths. Additionally, minor peaks located at 36.9° and 77.7° are ascribed to the (111) and (220) planes of CoO (JCPDS#43-1004), confirming the successful incorporation of CoO in the electrode [81,101]. No notable Mo-phase signal appeared in the XRD profiles, suggesting the absence of large Mo crystals, and the small amount of Mo is uniformly distributed in the Mo@CoO electrode. The uniform doping of Mo in CoO is beneficial for boosting its electronic conductivity, which facilitates efficient charge transfer and thus results in enhanced capacitive performance. Moreover, the surface information of the Mo@CoO-24 sample was examined using Raman spectroscopy, as shown in Figure S3. The peaks at 187, 467, 512, 602, and 670 cm^−1^ were attributed to the characteristic vibrations of CoO [102]. Moreover, the peaks located at 807 and 939 cm^−1^ corresponded to the symmetric stretching vibration of Mo=O bonds in MoO_3_ and the A_1_ mode of Mo=O, respectively [81,103]. The presence of these Mo-related vibrational peaks further reveals the successful doping of Mo.

**Figure 4:**
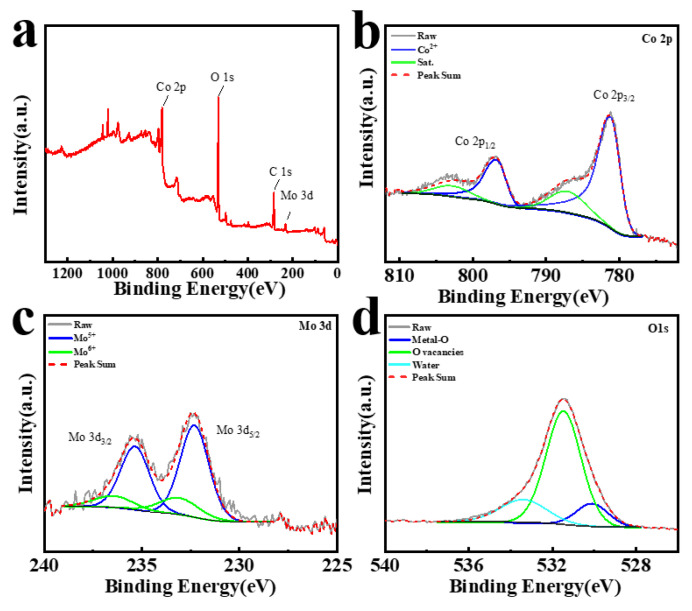
(**a**)Full XPS spectrum of Mo@CoO electrode, high-resolution XPS spectra of (**b**) Co 2p (**c**) Mo 3d, (**d**) O 1s spectrum of Mo@CoO electrode.

Thereafter, XPS detection was employed to analyze the surface content of Mo@CoO active materials. The XPS full survey spectrum presented in Figure 4a verifies that Co, Mo, O, and C are the main elements present in the Mo@CoO electrode. The deconvoluted Co 2p, Mo 3d and O 1s spectra are presented in Figure 4b, Figure 4c and Figure 4d, respectively. In Figure 4b, two prominent peaks located at positions 781.10 eV and 796.73 eV were accordingly indexed to Co 2p_3/2_ and Co 2p_1/2_ belonging to CoO, whereas the peaks positioned at 786.96 eV and 802.75 eV provided additional evidence for CoO formation [81,104]. In Figure 4c, the peaks appearing at 233.10 eV and 236.45 eV could be assigned to the Mo^6+^ 3d_5/2_ and Mo^6+^ 3d_3/2_ orbitals, whereas the peaks positioned at 232.30 eV and 235.35 eV were associated with the orbitals of Mo^5+^ 3d_5/2_ and Mo^5+^ 3d_3/2_, which confirmed the successful integration of Mo within the structure of CoO [81,105]. Furthermore, the peaks of 530.10 eV, 531.47 eV and 533.37 eV located at the O 1s spectrum of Mo@CoO could be deconvoluted to reveal the contributions of the Metal-O bond, oxygen vacancies, and surface-adsorbed water [106]. Therefore, the XPS analysis successfully revealed the introduction of molybdenum dopant into the structure of CoO and provides evidence for the presence of oxygen vacancies. Moreover, oxygen vacancies in Mo@CoO-24 were analyzed using room-temperature EPR (Figure S4). The EPR signal at g = 2.0060 closed to the free electron value of 2.0023, indicated that unpaired electrons had appeared, which furtherly confirmed the formation of oxygen vacancies in Mo@CoO-24 materials [65]. The simultaneous introduction of Mo doping and oxygen vacancies in CoO materials by EDC strategy was beneficial for the enhancement of the conductivity of the electrodes and energy storage [65,81].

**Figure 5:**
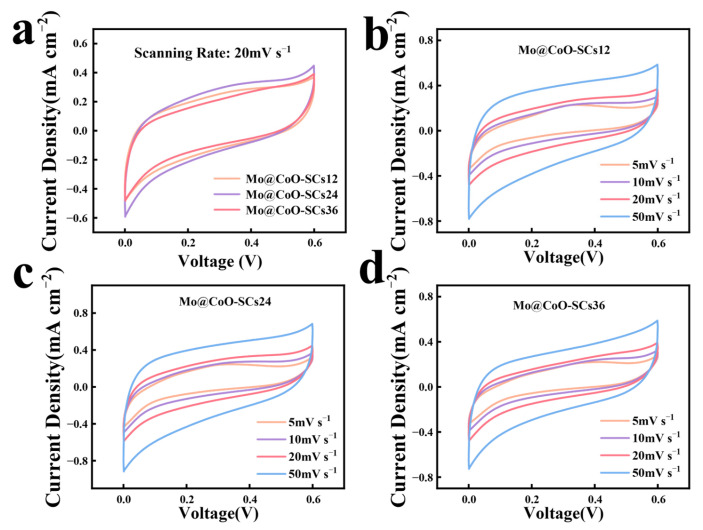
(**a**) CV profiles of Mo@CoO-SCs12, Mo@CoO-SCs24, and Mo@CoO-SCs36 at 20 mV s^−1^, CV profiles of (**b**) Mo@CoO-SCs12, (**c**) Mo@CoO-SCs24, and (**d**) Mo@CoO-SCs36 at various scan rates (5–50 mV s^−1^).

**Figure 6:**
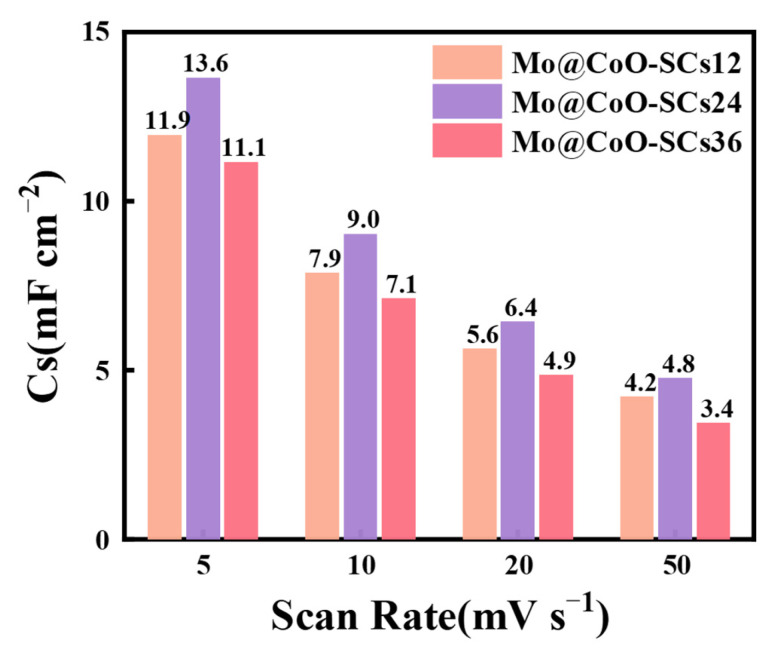
Corresponding areal capacitance of Mo@CoO-SCs12, Mo@CoO-SCs24, and Mo@CoO-SCs36 calculated from CV Profiles.

In Figure 5a, all the Mo@CoO-SCs12, Mo@CoO-SCs24, and Mo@CoO-SCs36 devices exhibited a quasi-rectangular CV shape at a scan rate of 20 mV s^−1^, indicating that good capacitive behavior was achieved for these devices fabricated by EDC. Among these three CV profiles, that of Mo@CoO-SCs24 demonstrated the largest CV curve area, whereas that of Mo@CoO-SCs36 showed the smallest CV curve area, suggesting that the largest capacitance and smallest capacitance was achieved by Mo@CoO-SCs24 and Mo@CoO-SCs36, respectively. The superior capacitive performance of Mo@CoO-SCs24 could be attributed to the optimized EDC pulse width of 24 μs, which facilitated the formation of a moderately sized, uniformly distributed oxide layer with a porous texture, as observed in SEM analysis. This morphology enhanced the active surface area, providing abundant sites for Faradaic redox reactions and improving electrolyte ion diffusion to active sites on the electrode. The inferior performance of Mo@CoO-SCs36 results from excessive particle aggregation reducing active surface area, while the sparse distribution of smaller particles in Mo@CoO-SCs12 limits active sites. Figure 5b–d further shows the CV profiles of Mo@CoO-SCs12, Mo@CoO-SCs24, and Mo@CoO-SCs36 at different scanning rates (5–50 mV s^−1^). The CVs for all the Mo@CoO samples prepared at various pulse widths maintained consistent quasi-box shapes, which further verified that good capacitive behavior was obtained for the Mo@CoO based binder-less devices fabricated by EDC strategy. Accordingly, Figure 6 presented the areal capacitance of Mo@CoO-SCs12, Mo@CoO-SCs24, and Mo@CoO-SCs36 fabricated by EDC. It could be observed that the devices of Mo@CoO-SCs12, Mo@CoO-SCs24, and Mo@CoO-SCs36 exhibited the highest capacitance of 11.9, 13.6 and 11.1 mF cm^−2^ at 5 mV s^−1^ respectively. The capacitance of Mo@CoO-SCs12, Mo@CoO-SCs24, and Mo@CoO-SCs36, respectively, maintained at 4.2, 4.8 and 3.4 mF cm^−2^ when the scan rate increased to 50 mV s^−1^. Moreover, it could be found that Mo@CoO-SCs24 prepared with a pulse width of 24μs obtained a maximum capacitance value, whereas the Mo@CoO-SCs36 fabricated with a pulse width of 36 μs gained the minimum capacitance value. This is because the large agglomeration of Mo@CoO particles prepared by a long pulse width of 36μs expressed significant limitation for ion access into the active materials. Moreover, the Mo@CoO active materials prepared with a short pulse width of 12 μs were sparsely covered on the cobalt current collector, which was not able to offer sufficient active sites for ions landing. In contrast, the Mo@CoO active materials synthesized with a medium pulse width of 24 μs depicted an even and porous distributed characteristic, which is extremely helpful for boosting the ion/electron transportation efficiency. Incidentally, we also measured samples prepared with a higher pulse width of 48 μs, as shown in Figure S5. It was found that the Mo@CoO device fabricated at 48 μs exhibited a relatively lower current response and shorter discharge time compared to those prepared at pulse widths of 12, 24, and 36 μs (Figure S5a,c). As a result, the Mo@CoO device prepared at a pulse width of 48 μs achieved a relatively lower capacitance value compared to those devices prepared at a pulse width of 12, 24 and 36 μs. Most importantly, the capacitance value obtained by Mo@CoO-SCs24 was superior to those of previously reported materials, such as expanded polystyrene/rGo fabricated through mixing and drying (11.9 mF cm^−2^) [107], graphene constructed supercapacitor prepared by sonication, filtration, spray coating, and layering (6.6 mF cm^−2^) [108], NiO/Co_3_O_4_ nanoparticles synthesized via drying, microwave irradiation, and the Staudenmaier method with stirring (5.2 mF cm^−2^) [109], and K_2_Ti_4_O_9_@Ni(OH)_2_/Ti produced by sonication, drying, and annealing (5.8 mF cm^−2^) [109]. The superior capacitance of Mo@CoO-SCs24 was due to the merit of the EDC technique, allowing for a binder-less integrated electrode design and the successful doping of the molybdenum element and oxygen vacancy in the CoO active materials.

**Figure 7:**
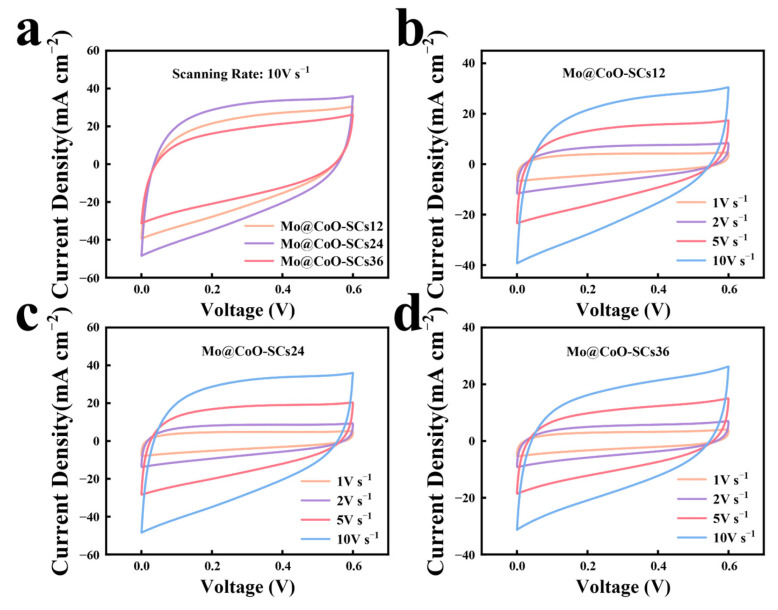
(**a**) CV profiles of Mo@CoO-SCs12, Mo@CoO-SCs24, and Mo@CoO-SCs36 at ultrahigh scan rate of 10 V s^−1^, CV curves for (**b**) Mo@CoO-SCs12, (**c**) Mo@CoO-SCs24, and (**d**) Mo@CoO-SCs36 at various ultrahigh scan rates (1–10 V s^−1^).

**Figure 8:**
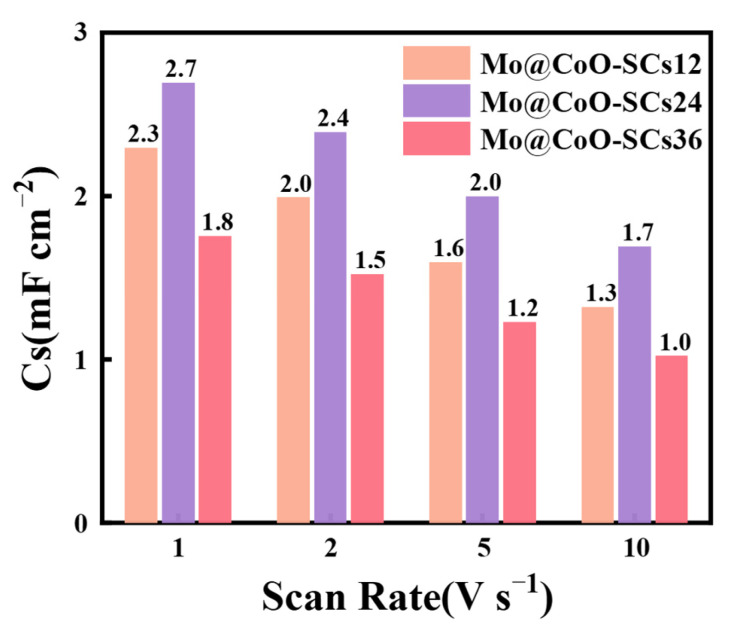
Corresponding areal capacitance of Mo@CoO-SCs12, Mo@CoO-SCs24, and Mo@CoO-SCs36 calculated from the ultrahigh CV Profiles.

Moreover, aiming to further demonstrate the merit of EDC for the preparation of Mo@CoO based coin-cell supercapacitors to meet the requirement of industrial applications, ultrahigh scan rates of 1, 2, 5 and 10 V s^−1^ were applied to check the capacitive performance of Mo@CoO-SCs12, Mo@CoO-SCs24 and Mo@CoO-SCs36 coin-cell devices. Figure 7b–d depict that all the CV profiles of Mo@CoO-SCs12, Mo@CoO-SCs24, and Mo@CoO-SCs36 at super-high scan rates of 1–10 V s^−1^ exhibited a quasi-rectangular shape, indicating that good capacitive behavior was achieved for these devices prepared by EDC. As the scan rate increases, the electrochemical reactions on the electrode surface accelerate, resulting in higher current responses, thereby increasing the CV curve area. Furthermore, the good capacitive performance of Mo@CoO-SCs demonstrated at high scan rates indicates their excellent dynamic response and suitability for high-rate energy storage applications. It is notable that the Mo@CoO-SCs demonstrated superior high-rate performance even at 10 V s^−1^, which is 200 times higher than typical scan rates applied for reported CoO electrodes [89,110,111]. The good capacitive performance achieved for all these devices is due to the Mo@CoO based binder-less integrated electrode design decorated with the doping of the molybdenum element and oxygen vacancy, which is greatly beneficial for the enhancement of their ionic/electronic conductivity. Moreover, it can be seen from Figure 7a that Mo@CoO-SCs24 device showed the largest CV curve area whereas Mo@CoO-SCs36 device obtained the smallest CV curve area, indicating that Mo@CoO-SCs24 obtained the largest current response and best capacitive performance and Mo@CoO-SCs36 gained the smallest current response and the worst capacitive performance among these three devices. This phenomenon was furtherly proved by their calculated areal capacitance presented at the bar graph of Figure 8. The results indicate that the areal capacitance decreases as the scan rate increases, as shown in the equation

i = CdU/dt

where i is the current, C is the capacitance, and dU/dt represents the rate of voltage change (scan rate). However, at higher scan rates, ions in the electrolyte have insufficient time to penetrate the inner pores of the electrode material, reducing charge storage on the electrode surface. As a result, only the outermost active sites contribute to capacitance, leading to the observed areal capacitance decrease. The Mo@CoO-SCs24 device obtained a capacitance of 2.7 mF cm^−2^ (1 V s^−1^), which is 1.17 and 1.5 times greater than that presented by Mo@CoO-SCs12 and Mo@CoO-SCs36, respectively. When the scan rate increased to 10 V s^−1^, the Mo@CoO-SCs24 device obtained a capacitance of 1.7 mF cm^−2^, which is 1.31 and 1.70 times higher than that of Mo@CoO-SCs12 and Mo@CoO-SCs36, respectively. This indicates that the capacitance gap between Mo@CoO-SCs12, Mo@CoO-SCs24 and Mo@CoO-SCs36 became lager at 10 V s^−1^.

Additionally, Figure S6 illustrates the cycling stability of Mo@CoO-SCs12, Mo@CoO-SCs24, and Mo@CoO-SCs36 devices tested at a scan rate of 500 mV s^−1^ over 2000 cycles. The results reveal that Mo@CoO-SCs24 exhibits minimal capacitance decay, with a capacitance retention rate of 97.07%, significantly outperforming Mo@CoO-SCs12 and Mo@CoO-SCs36, which show retention rates of 65.39% and 54.55%, respectively. The post-cycling SEM images are presented in Figure S7. Compared to the pre-cycling images, no significant morphological changes were observed, indicating the structural stability of the Mo@CoO electrodes throughout the cycling process. The chemical composition of the electrode surfaces was analyzed using EDS, as summarized in Table S1. The Mo@CoO-24 electrode exhibits the highest Mo content (3.68 at.%) among all samples, reflecting efficient Mo doping achieved through the EDC process, and its correlation with enhanced electrochemical performance. Moreover, the atomic ratio of Co and Mo for Mo@CoO-12, Mo@CoO-24 and Mo@CoO-36 before cyclic testing was 24.40%, 22.91% and 36.97%, respectively. However, these atomic ratio decreased to 15.43%, 20.99%, and 28.37% accordingly after the cyclic testing, which is attributed to the partial corrosion of the electrodes. As shown in Figure S8, the internal resistance of the three devices before and after cycling was measured through EIS and simulated with the equivalent circuit model [112,113,114,115,116]. The analysis of Nyquist plots for the EIS data at different frequencies before and after cycling showed very small variation of resistance and capacitance Mo@CoO-SCs12 and Mo@CoO-SCs36 devices. The data for Mo@CoO-SCs24 sample showed a decrease in the real part of the impedance, which indicated lower resistance, and an increase in the imaginary part, which indicated a decrease in capacitance. The EIS data were analyzed using an equivalent circuit, where the capacitive component was presented by the constant phase element. The experimental data were in good agreement with the simulation results.

**Figure 9:**
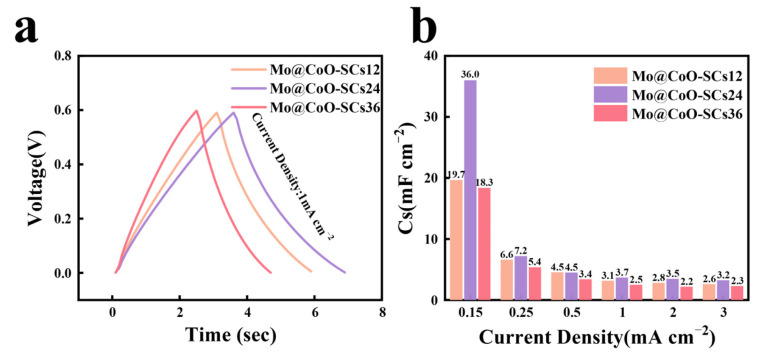
(**a**) GCD profiles of Mo@CoO-SCs12, Mo@CoO-SCs24, and Mo@CoO-SCs36 at 1 mA cm^−2^, (**b**) corresponding to areal capacitance of Mo@CoO-SCs12, Mo@CoO-SCs24, and Mo@CoO-SCs36 calculated from GCD Profiles at different current densities (0.15–3 mA cm^−2^).

Moreover, GCD detection was conducted to further investigate the capacitive performance of Mo@CoO-SCs12, Mo@CoO-SCs24, and Mo@CoO-SCs36. It was observed that all the coin-cell devices of Mo@CoO-SCs12, Mo@CoO-SCs24 and Mo@CoO-SCs36 achieved a symmetric GCD profile, which further proved that good capacitive performance was achieved for the devices fabricated by the EDC strategy. In addition, the Mo@CoO-SCs24 device illustrated the longest discharge time, whereas Mo@CoO-SCs24 obtained the shortest discharge time, which further confirmed that Mo@CoO-SCs24 prepared with a pulse width of 24 μs by EDC achieved optimum capacitive performance. The corresponding areal capacitances derived from the GCD profiles at various current densities (0.15–3 mA cm^−2^) are presented at Figure 9b. The Mo@CoO-SCs24 showed an areal capacitance of 36.0 mF cm^−2^ at 0.15 mA cm^−2^, which is 1.83 times and 1.97 times greater than that presented by Mo@CoO-SCs12 (19.7 mF cm^−2^) and Mo@CoO-SCs36 (18.3 mF cm^−2^), respectively. The obtained areal capacitance of Mo@CoO-SCs24 surpasses several previously reported cobalt-based materials (see Table S2), such as CuCo_2_O_4_ (10.88 mF cm^−2^) fabricated by laser scribing, annealing, brush coating, and hydrothermal synthesis [117]; Co_3_O_4_@C (20.03 mF cm^−2^) prepared via electrospinning and calcination [118]; Co(OH)_2_ (5.26 mF cm^−2^ and 0.0505 mF cm^−2^) obtained by electrodeposition [119,120]; Co₃O₄ and Co(OH)_2_ (35.68 mF cm^−2^) synthesized through a one-pot hydrothermal method, followed by stirring, vacuum filtration, drying, ultrasonication, and inkjet printing [121]; and Co_3_O_4_@MnO_2_ (13.9 mF cm^−2^) prepared by solvothermal synthesis, ultrasonication, dip-coating, and dispersion techniques [122]. This phenomenon, obtained by GCD testing, is consistent with the CV results illustrated in Figure 6 and Figure 8. This is because severe agglomeration of Mo@CoO particles prepared with a long pulse width of 36 μs expressed a significant limitation for ion access into the active materials. Moreover, the Mo@CoO particles prepared with a short pulse width of 12 μs were sparsely covered on the cobalt current collector, which was not able to offer a sufficient number of active sites for ion landing. In contrast, the Mo@CoO active materials synthesized with a medium pulse width of 24 μs depicted an even and porous distributed characteristic, which is extremely helpful for boosting ion/electron transportation efficiency.

Therefore, it could be concluded that binder-less molybdenum doped CoO integrated electrodes, one-step fabricated using a simple EDC method, enable rapid synthesis of Mo@CoO active materials with an oxygen vacancy directly onto cobalt substrates, without the need for pre-made templates, conductive additives, or chemicals. Most importantly, the EDC method enables precise control over the discharge processing parameter of pulse width, which facilitates the tailoring of the surface morphologies of the as-prepared Mo@CoO active materials, thus directly controlling the electrochemical performance of the supercapacitors. These results demonstrate that this novel EDC strategy showed great potential for efficiently fabricating various metal oxide-based binder-less integrated electrodes for various applications, like supercapacitors, batteries and sensors.

## 4. Conclusions

Herein, binder-less Mo@CoO electrodes were successfully fabricated via a simple EDC method, which facilitates tailoring the surface morphologies of Mo@CoO by EDC pulse width. It was found that the fabricated Mo@CoO-SCs24 achieved a maximum areal capacitance of 36.0 mF cm^−2^ (0.15 mA cm^−2^), which is 1.83 and 1.97 times higher than that of Mo@CoO-SCs12 and Mo@CoO-SCs36. This is because the Mo@CoO active materials synthesized with a medium pulse width of 24 μs depicted an even and porous distribution characteristic, which is extremely helpful for boosting ion/electron transportation efficiency. In contrast, Mo@CoO particles prepared by a long pulse width of 36 μs obtained severe agglomeration, and Mo@CoO particles prepared with a short pulse width of 12 μs were sparsely covered on the cobalt current collector, which was not able to offer efficient ionic/electronic conductive property for energy storage. However, the current work faces the challenge of synthesizing foreign element doped metal oxides films with high areal capacitance. Overcoming this obstacle is critically important for unlocking the full potential of the EDC strategy and opening a new window for efficient fabrication of various metal oxide based next-generation energy storage devices with high energy density.

## Data Availability

Data are contained within the article.

## Supplementary Materials

The following supporting information can be downloaded at: https://www.mdpi.com/article/10.3390/ma18010080/s1, Figures S1. SEM image of raw Co metal substrate. Figure S2. XRD patterns of Mo@CoO samples with different pulse widths and the bare Co substrate. Figure S3. Raman spectroscopy of Mo@CoO-24. Figure S4. Room-temperature EPR spectra of Mo@CoO-24. Figure S5. (a) CV profiles of Mo@CoO-SCs12, Mo@CoO-SCs24, Mo@CoO-SCs36 and Mo@CoO-SCs48 at 20 mV s^−1^, (b) Corresponding areal capacitance of Mo@CoO-SCs with different pulse widths calculated from CV Profiles, (c) GCD profiles of Mo@CoO-SCs12, Mo@CoO-SCs24, Mo@CoO-SCs36 and Mo@CoO-SCs48 at 1 mA cm^−2^, and (d) corresponding areal capacitance of Mo@CoO-SCs with different pulse widths calculated from GCD Profiles. Figure S6. Cycle stability testing of (a) Mo@CoO-SCs12, (b) Mo@CoO-SCs24, and (c) Mo@CoO-SCs36. Figure S7. The post-cycling SEM images of (a) Mo@CoO-12, (b) Mo@CoO-24, and (c) Mo@CoO-36. Figure S8. EIS Nyquist plots of Mo@CoO-SCs12, Mo@CoO-SCs24, and Mo@CoO-SCs36: (a) before cycling and (b) after cycling. Table S1. Chemical composition results of samples examined by EDS. Table S2. Several previously reported Co-based materials electrodes for supercapacitor applications compared with this work.
