# Supplementary material for: Binder-Less Molybdenum Doped CoO Based Integrated Electrodes Fabricated by Electric Discharge Corrosion for High-Efficiency Supercapacitors
Source: Materials (Basel). 2024 Dec 27;18(1):80. doi: 10.3390/ma18010080 (PMC11721405; doi:10.3390/ma18010080)
Supplement: Supplementary file 1 [file materials-18-00080-s001.zip › materials-3367717-supplementary.pdf]

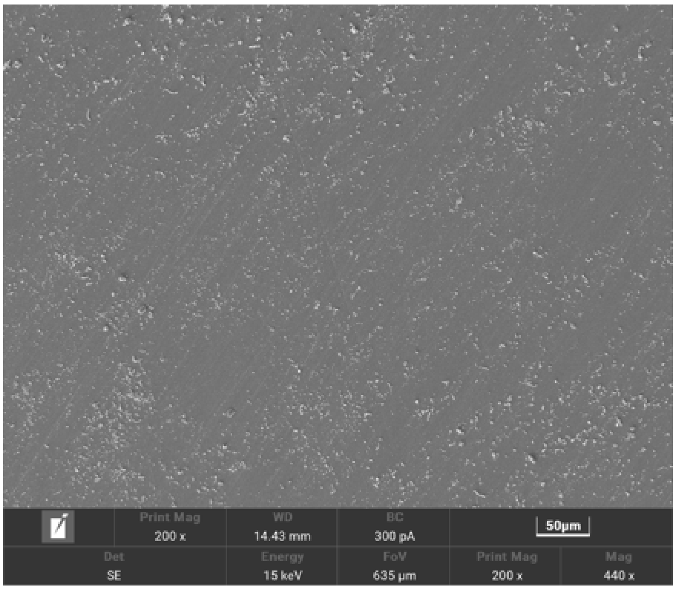

Figure S1. SEM image of raw Co metal substrate.

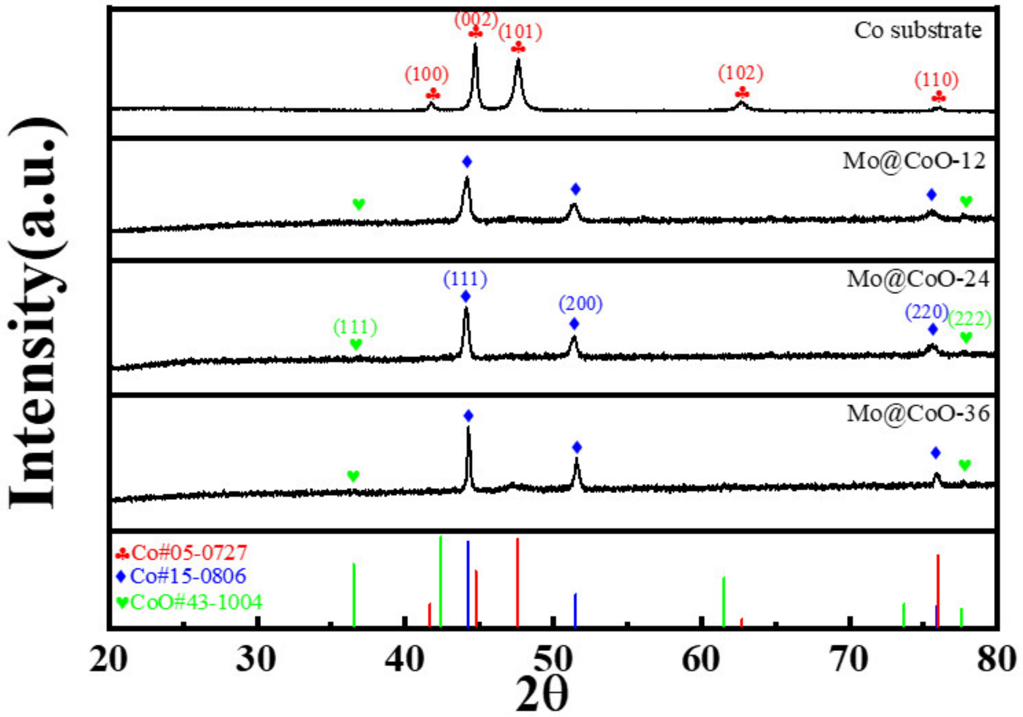

Figure S2. XRD patterns of Mo@CoO samples with different pulse widths and the bare Co substrate.

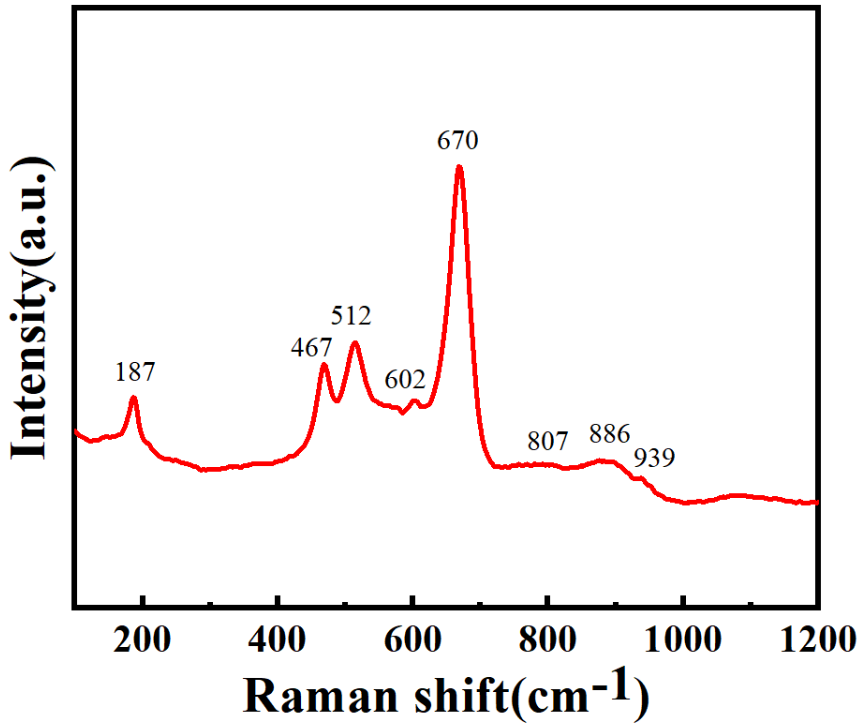

Figure S3. Raman spectroscopy of Mo@CoO-24.

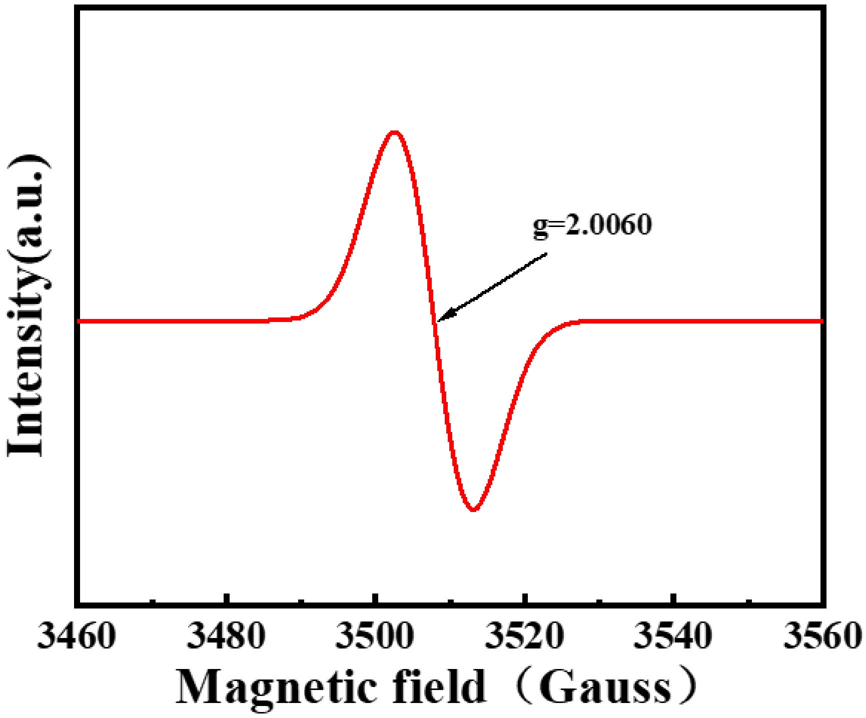

Figure S4. Room-temperature EPR spectra of Mo@CoO-24.

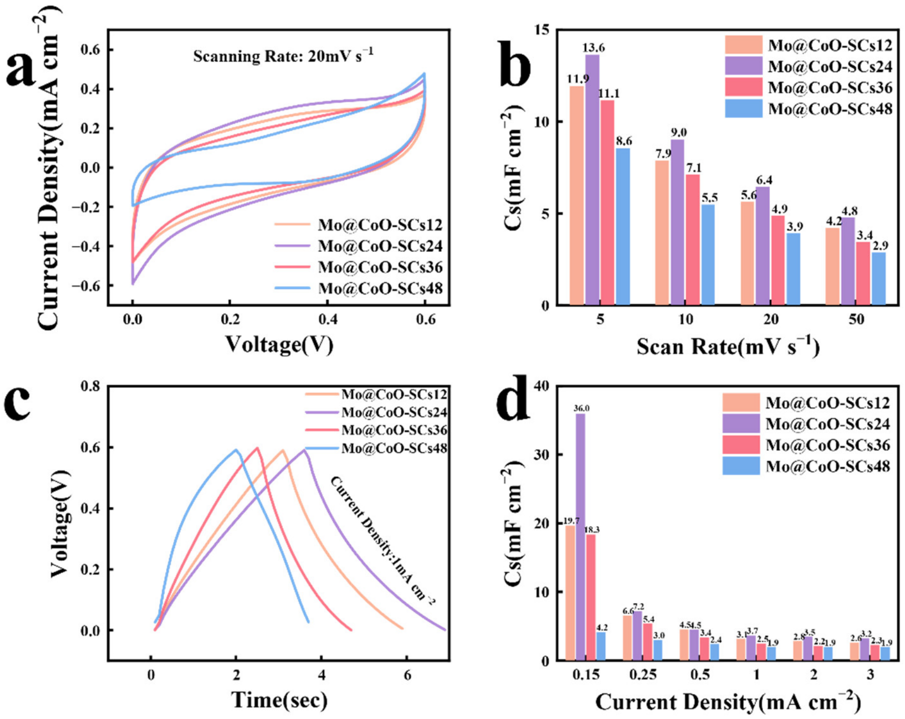

Figure S5. (a) CV profiles of Mo@CoO-SCs12, Mo@CoO-SCs24, Mo@CoO-SCs36 and Mo@CoO-SCs48 at 20 mV s^{-1}, (b) Corresponding areal capacitance of Mo@CoO-SCs with different pulse widths calculated from CV Profiles, (c) GCD profiles of Mo@CoO-SCs12, Mo@CoO-SCs24, Mo@CoO-SCs36 and Mo@CoO-SCs48 at 1 mA cm^{-2}, and (d) corresponding areal capacitance of Mo@CoO-SCs with different pulse widths calculated from GCD Profiles.

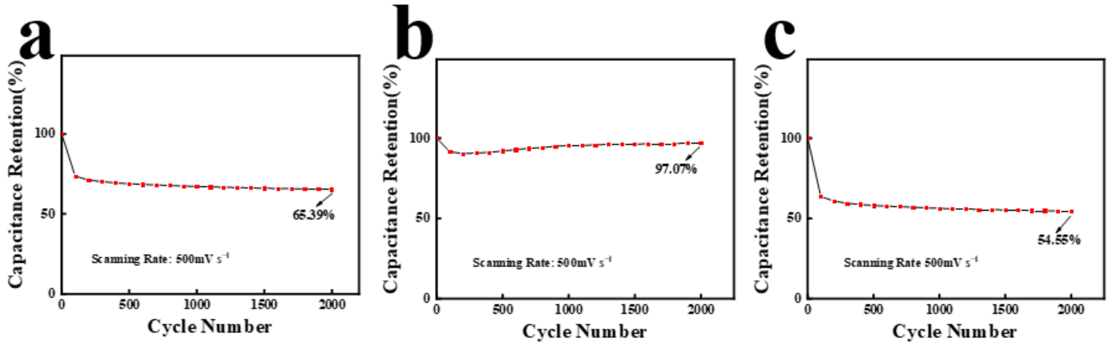

Figure S6. Cycle stability testing of (a) Mo@CoO-SCs12, (b) Mo@CoO-SCs24, and (c) Mo@CoO-SCs36.

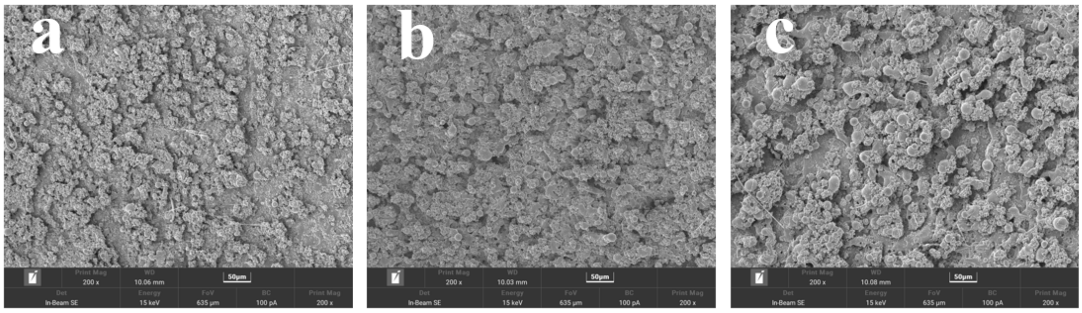

Figure S7. The post-cycling SEM images of (a) Mo@CoO-12, (b) Mo@CoO-24, and (c)

Mo@CoO-36.

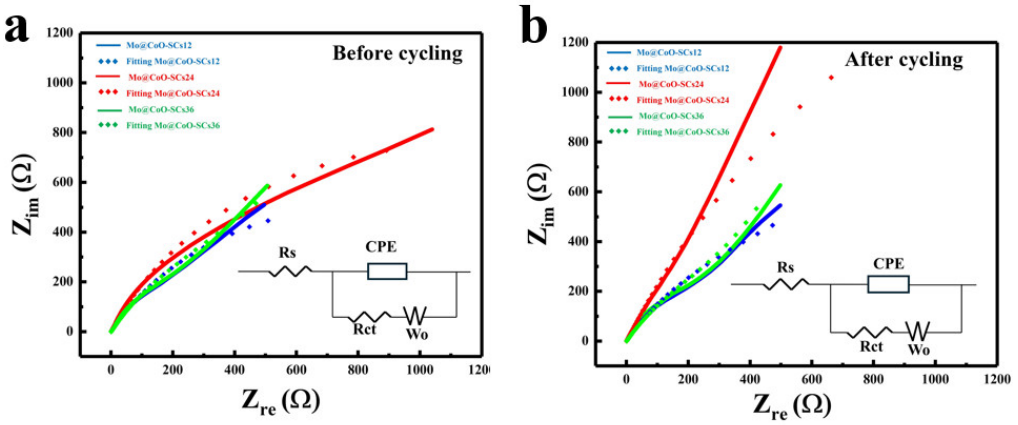

Figure S8. EIS Nyquist plots of Mo@CoO-SCs12, Mo@CoO-SCs24, and Mo@CoO-SCs36: (a) before cycling and (b) after cycling.

Table S1. Chemical composition results of samples examined by EDS.

Sample	Co (at.%)	Mo (at.%)	Atomic ratio of Co and Mo
Mo@CoO-12	84.44	3.46	24.40
Mo@CoO-24	84.31	3.68	22.91
Mo@CoO-36	86.87	2.35	36.97
Mo@CoO-12-after cycling	69.17	4.48	15.43
Mo@CoO-24-after cycling	65.07	3.10	20.99
Mo@CoO-36-after cycling	77.74	2.74	28.37

Table S2. Several previously reported Co-based materials electrodes for supercapacitor applications compared with this work.

Supercapacitor materials	Methods	Current collectors	Capacitance (mF cm^{-2})	References
$CuCo_2O_4$	laser scribing, annealing, brush coating, and hydrothermal synthesis	Flexible PET	10.88	[1]
Co_3O_4@C	electrospinning and calcination	Carbon cloth	20.03	[2]
$Co(OH)_2$	Electrodeposition	FTO	5.26	[3]
$Co(OH)_2$	Electrodeposition	Au-PET	0.0505	[4]
Co_3O_4 and $Co(OH)_2$	one-pot hydrothermal method, followed by stirring, vacuum filtration, drying, ultrasonication, and inkjet printing	silver nanowire networks	35.68	[5]
Co_3O_4@MnO_2	solvothermal synthesis, ultrasonication, dip-coating, and dispersion technique	Ni wire	13.9	[6]
Mo@CoO	EDC	Co	36.0	This work

Reference

1. A. Basu, M. Bhardwaj, Y. Gawli, C. Rode, S. Ogale, A Robust Highly Flexible All–solid–state Micro Pseudocapacitor Based on Ternary Oxide CuCo2O4 having Ultrathin Porous Nanowall Type Morphology Blended with CNT, ChemistrySelect 1(16) (2016) 5159-5164.
2. R. Barik, A. Raulo, S. Jha, B. Nandan, P.P. Ingole, Polymer-derived electrospun Co3O4@ C porous nanofiber network for flexible, high-performance, and stable supercapacitors, ACS Applied Energy Materials 3(11) (2020) 11002-11014.
3. S. Kalasina, P. Pattanasattayavong, M. Suksomboon, N. Phattharasupakun, J. Wutthiprom, M. Sawangphruk, A new concept of charging supercapacitors based on the photovoltaic effect, Chemical Communications 53(4) (2017) 709-712.
4. Y. Tian, J. Gong, W. Zhu, Vertically-aligned Co (OH) 2 Nanosheet Films for Flexible All-solid-state Electrochemical Supercapacitor, IOP Conference Series: Earth and Environmental Science, IOP Publishing, 2017, p. 012131.
5. N. Afsahi, M. Majumder, N. Naseri, Printed flexible solid-state microsupercapacitor with highly-stable aqueous cobalt-based inks, Chemical Engineering Journal 493 (2024) 152356.
6. X. Niu, G. Zhu, Z. Yin, Z. Dai, X. Hou, J. Shao, W. Huang, Y. Zhang, X. Dong, Fiber-based all-solid-state asymmetric supercapacitors based on Co 3 O 4@ MnO 2 core/shell nanowire arrays, Journal of Materials Chemistry A 5(44) (2017) 22939-22944.
